# Omics-aided design genome editing strategy for challenging human immortalized cell models

**DOI:** 10.1371/journal.pone.0341124

**Published:** 2026-02-12

**Authors:** Patricia Mendoza-Garcia, Benjamin Keith, Markus Nordberg, Ella Quist, Cristina Ferrás, Ghaith M. Hamza, Ramy Elgendy, Stephanie Kay Ashenden, Jordi Chi, Natalie R. van Zuydam, Neil Hattersley, Xiang Zhang

**Affiliations:** 1 Assays, Profiling & Cell Sciences, Discovery Sciences, BioPharmaceuticals R&D, AstraZeneca, Gothenburg, Sweden; 2 Data Sciences & Quantitative Biology, Discovery Sciences, BioPharmaceuticals R&D, AstraZeneca, Gothenburg, Sweden; 3 Chemical Biology & Proteomics, Discovery Sciences, BioPharmaceuticals R&D, AstraZeneca, Waltham, United States of America; 4 Center for Genomics Research, Discovery Sciences, BioPharmaceuticals R&D, AstraZeneca, Gothenburg, Sweden; 5 Translational Sciences and Clinical Development, Early CVRM, BioPharmaceuticals R&D, AstraZeneca, Cambridge, United Kingdom; 6 Protein Structure & Biophysics, Discovery Sciences, BioPharmaceuticals R&D, AstraZeneca, Gothenburg, Sweden; Northwestern University Feinberg School of Medicine, UNITED STATES OF AMERICA

## Abstract

CRISPR-Cas9 has become a popular genome editing tool for biomedical research and drug development due to its capability to enable precise correction or integration of genetic mutations in the genome. However, precise genome editing competency varies dramatically between cell types depending on their capabilities for DNA damage. In this proof-of-concept study, we took the example of HepG2 and MCF7 to show that omics profiling identifies bottlenecks that are associated with poor precise knock-in (KI) efficiency in hard-to-engineer cells. These bottlenecks include previously described factors such as the predominance of non-homologous end joining (NHEJ) repair and impaired homologous recombination (HR) capability, but also reveals apoptotic priming status of the cells as a limiting factor. Upon further comparative analysis between HepG2 and MCF7 cells, we pinpointed and validated the proliferating cell nuclear antigen (PCNA) as a target to overexpress to enhance precise KI efficiency in MCF7. Overall, we describe how employing a multi-omics approach to characterize cell models of interest can facilitate an in-depth understanding of their editability molecular signature, empowering us to manipulate the activity of key pathways for precise editing, and therefore increase efficiency of desired editing outcomes.

## Introduction

There is an increasing demand for precise genome editing due to their potential for drug discovery and therapeutic applications, such as disease modeling, functional genomics, and development of cell therapies. For this purpose, Clustered Regularly Interspaced Short Palindromic Repeats associated protein 9 (CRISPR-Cas9) or related technologies are deployed to generate these edits. However, the likelihood of successfully achieving precise genome edits (including knock-out and knock-in) varies widely and often acts as a limiting factor. When performing KI precise edits with CRISPR/Cas9 systems, a major factor affecting the observed variation is the underlying competency of the target cell to respond to the editing challenge and repair the induced DNA lesion via DNA damage response (DDR) activation. In mammalian cells, double strand breaks (DSBs) are primarily repaired by three DNA repair mechanisms: non-homologous end joining (NHEJ), microhomology-mediated end joining (MMEJ), and homologous recombination (HR) [1]. While NHEJ and MMEJ are inherently error prone mechanisms, HR resolves DNA lesions in an error-free manner, and therefore it is desired for CRISPR-Cas9 precise editing. These precise KI edits require the introduction of DSBs at a specific locus and repair of the DNA lesion by HR. However, the DNA repair mechanism of choice varies depending on a number of factors such as cell cycle phase or activity and kinetics of different DNA repair pathways. While NHEJ is active throughout the entire cell cycle, MMEJ and HR are restricted to S and G2 phases respectively [2]. In addition, HR is a much slower process compared to NHEJ, since it requires extensive DNA end resection to resolve DNA lesions [3,4].

The NHEJ dominance is an obvious bottleneck for precise KI efficiency, and previous studies have focused on studying the impact on modulating DDR proteins, such as inhibiting NHEJ factors [5–7] and stimulating DNA end resection [8]. Although these strategies showed great potential in individual cases, they have not been able to be generalized across different cell types. The reason is that apart from NHEJ dominance, different cells can have different HR capabilities. A heuristic evaluation of HR capability can be done through the HR deficiency (HRD) score [9]. HRD scores are calculated as the sum of three independent DNA-based measures of genomic instability: loss of heterozygosity (LOH), telomeric allelic imbalance (TAI), and large-scale transitions (LST). HRD score inversely correlates with competence to execute homologous recombination, so that cell types with HRD scores above 42 are considered HR deficient [9]. Although cells with different HR repair status were reported to display different gene expression patterns [10], it is not clear whether the HRD scores indeed reflect phenotypic differences in HR related genes.

Recent developments in omics technologies, such as next generation sequencing and mass spectrometry, allow us to measure the expression and abundance of thousands of genes and proteins in an unbiased manner. This is particularly useful for characterizing cell-specific CRISPR-induced DDR, which involves a complex network of many different and interconnected proteins [11]. In this study we aim to explore how leveraging omics profiling can enhance our understanding of improving the efficiency of precise KI events. As a proof-of-concept, we focused on two cell lines with distinct HRD scores: HepG2 and MCF7. To assess whether the HRD scores indeed reflect phenotypic or abundance differences in HR related genes, we performed global proteomics on HepG2 and MCF7 at baseline. In addition, to gain insights into the CRISPR-induced DDR in these two cell types, we performed transcriptomics analysis following electroporation of CRISPR editing reagents targeting *SERPINA1* locus (here considered safe-to-edit locus as is not required for cell viability). Our integrated omics analysis confirmed NHEJ dominance in both cell types and identified two additional bottlenecks in the HR-deficient MCF7 cells impeding repair of Cas9-derived cuts and exogenous DNA integration: 1) low abundance of HR related proteins; 2) apoptotic priming state prior to p53 activation. Based on these findings, our omics analysis identified a list of DDR proteins amenable to modulation to potentially improve KI efficiency. Among them we identified the DNA replication factor PCNA, whose overexpression enhanced precise KI efficiency by 40% in MCF7, in combination with NHEJ and MMEJ small molecule inhibitors. Overall, we demonstrate the potential of employing multi-omics approaches to interrogate and modulate the molecular signatures of immortalized cell models to successfully engineer them.

## Method & materials

### Study design

In this study we applied an omics profiling approach to investigate CRISPR-induced DDR in HepG2 (HRD score = 9) and MCF7 (HRD score = 61) [12]. Both cell types were electroporated with the same CRISPR ribonucleoprotein (RNP) complex (see “Transfections” section) and cultured in separate plates to avoid cross-contamination. RNA and protein samples were collected 24 and 48 hours after electroporation. As a result, there are two hard-to-change factors in this study. To be able to compare between different cell lines and time points, we applied the split plot design, in which each plate is considered as a whole plot.

### Plasmid

Protein sequences for PCNA and EGFP overexpression plasmids were retrieved from UniprotKB (PCNA #P12004, EGFP #C5MKY7) and back translated. Codon optimized sequences were synthesized by GeneArt (ThermoFisher) and assembled into pMAZ-T backbone (AZ proprietary) downstream of a CMV promoter using GoldenGate assembly (NEB). The sequence fidelity of cloned constructs was confirmed through Sanger-sequencing (Genewiz/Azenta).

### CRISPR reagents

For cell engineering purposes to specifically address precise KI efficiency, we used synthetic sgRNAs targeting *SERPINA1* locus in combination with a single-strand DNA (HDR template) consisting of 39 nt to be integrated, flanked by 30 nt homology arms. Synthetic sgRNAs and HDR template were synthesized by Sigma-Aldrich, MERCK. Spacers and donor sequences are summarized in S1 Table. The following small molecules were used in this study. PolQi1 (WO2021/028643, Example 158), PolQi2 (WO2020/243459, Example 99) were provided by AstraZeneca (Gothenburg, Sweden), and AZD7648 (HY-111783, MedChemExpress) is commercially available. All compounds were dissolved in DMSO (Sigma-Aldrich, MERCK #D2650) at a concentration of 10 mM.

### Cell culture

MCF7 cells (ATCC HTB-22) were maintained in RPMI 1640 Medium, GlutaMAX, HEPES (Gibco #72400−047) with 10% FBS (Gibco #10270−106). HepG2 (ATCC HB-8065) were cultured in MEM, GlutaMAX (Gibco #41090−036) with 1x MEM non-essential amino acids solution (Gibco #11140−050), 1% (v/v) sodium pyruvate (Gibco #11360−070), and 10% FBS (Gibco #10270−106). Cell lines were regularly tested for mycoplasma contaminations, and cell identity was confirmed through STR profiling.

### Transfections

#### Transfections – RNP preparation and delivery.

To prepare RNP complexes, spCas9-GFP (Sigma, MERCK #CAS9GFPPRO), sgRNAs, and ssDNA HDR template were incubated in a [1:1:1 molar ratio] for 10–15 min at room temperature. RNPs were kept on ice until electroporation. During RNP incubation, cells were washed with PBS (Gibco #10010023), detached with TrypLE Express Enzyme (Gibco #12604013), resuspended in cell culture medium, and cell number and viability were assessed using Nucleocounter NC-3000 cell counter (ChemoMetec). Prior to electroporation, cells were transferred into 15 ml tubes, washed with PBS and resuspended in Buffer R (Neon Transfection System, Invitrogen, #MPK1025) to a final density of 1 x 10^7^ cells/ml. For electroporation, 5 x 10^5^ cells (MCF7 or HepG2) were combined with 60 pmol spCas9 nuclease, 60pmol sgRNA (30pmol per guide), and 60pmol ssDNA. Electroporation was performed using Neon system (Invitrogen) applying the following settings: 1600V, 10ms pulse, 3 pulses. Afterwards, cells were transferred into 24-well plate format containing cell culture media with DMSO or small molecules at different concentrations (PolQi1 and PolQi2 at 1.5 µM, AZD7648 at 1µM).

#### Transient transfections – plasmid delivery.

MCF7 cells were seeded into T75 flasks (60,000 cells/cm^2^) 24 hours before transfections. pMAZ-CMV-PCNA plasmid was transfected using Lipofectamine 3000 transfection reagent (Invitrogen, #L3000001) following manufacturer’s instructions. Briefly, a total of 8 µg of plasmid DNA was used to transfect cells in T75 format. As positive control, the same number of cells were transfected in T75 flasks using pMAZ-CMV-EGFP, to address transfection efficiency and CMV promoter activity. Cells were cultured for 48h before the editing experiments.

### Deep-targeted amplicon next-generation sequencing

Genomic DNA was extracted at two timepoints: 24 and 48h after electroporation of CRISPR reagents. Cells were washed once with PBS and DNA extracted with QuickExtract (Lucigen #QE09050). Cells in QuickExtract were transferred to PCR tubes and incubated for 10 min at 70 °C followed by 10 min at 98 °C for enzyme deactivation. Cell lysates were stored at −20 °C until library preparation. Deep-targeted amplicon sequencing was performed from genomic DNA using the NextSeq platform (Illumina). In brief, 1–3 µL genomic DNA from QE, or 50 ng of purified genomic DNA, was used to generate amplicons flanking the CRISPR edited sites with two sequential rounds of PCR. In the first round of PCR, forward and reverse sequencing adaptors were introduced with the amplicon-specific primers (S1 Table). Amplicons were generated with Phusion Flash High-Fidelity PCR Master Mix (Thermo Fisher Scientific, #F548L) in a 15 µL reaction containing 250 nM of target-specific primers using the following cycling conditions: 98 °C for 3 min, 30-35x (98 °C for 10 s, 60 °C for 5 s, 72 °C for 5 s). PCR products were bead-purified using HighPrep PCR Clean-up System (Magbio Genomics, #AC60050) and analyzed on a fragment analyser (Agilent) to determine amplicon size and concentration. 0.5 ng of PCR1 product was subjected a second round of PCR to add unique Illumina indexes (Nextera XT Index Kit, Illumina, #FC-131–1096) with KAPA Hifi Hotstart Ready Mix (Roche, #07958927001) in a 25 µL reaction including 500 nM indexing primers. Thermocycling conditions were: 72 °C for 3 min, 98 °C for 30 s, 10x (98 °C for 10 s, 63 °C for 30 s, 72 °C for 3 min), 72 °C for 5 min. Purity and average length were analyzed with fragment analysis (Agilent), and concentration was quantified with a QuBit 4 Fluorometer (QuBit dsDNA HS Assay Kit, Life Technologies, Thermo Fisher Scientific). DNA libraries were sequenced on Illumina NextSeq 500 or Illumina MiSeq platforms.

### RNA sequencing

Total RNA was isolated from 5 × 10⁶ cells using a lysis buffer mix containing Proteinase K from the RNAdvance Tissue kit (Beckman Coulter). Cells were vortexed thoroughly and incubated at 37°C for 25 minutes. RNA isolation, including a 15-minute DNase I treatment, was carried out according to the manufacturer’s protocol on a Biomek i7 Hybrid robotic workstation (Beckman Coulter). RNA quantity and quality were assessed using a Fragment Analyzer 5300 system (Agilent), and RNA integrity number (RIN) values were recorded. Total RNA-seq libraries were prepared using the KAPA RNA HyperPrep with RiboErase kit (Roche) on a Tecan Fluent® liquid handler, following the manufacturer’s instructions. Library integrity and quality were evaluated with the SS NGS Fragment Kit (1–6000 bp; Agilent) on a fragment analyzer. Sequencing was performed as paired-end 2 × 100 bp on an Illumina NovaSeq 6000 platform using a v1.5 SP Reagent kit (Illumina).

### Proteomics data generation

Cell pellets were lysed through heating, and proteins were reduced and alkylated using the one pot buffer PreOmics iST kit (PreOmics, # P.O.00027). Briefly, lysis buffer was added directly to the cell pellet and incubated at 95 °C for 10 min for cell lysis, reduction, and alkylation of proteins. Cell lysates were normalized using BCA assay (ThermoFisher, #23225), and 50 µg from each condition was subjected to enzymatic proteolysis into peptides using trypsin:LysC (1:50 [wt/wt] enzyme:protein ratio) via overnight digestion. Subsequently, peptides were cleaned using a styrene-divinylbenzene based sorbent (PreOmics, # P.O.00027). Purified peptides were vacuum-centrifuged to dryness and reconstituted in double-distilled water with 2 vol% acetonitrile and 0.1 vol% formic acid for single-run LC-MS analysis. Peptides were loaded onto EvoTips (EvoSep Biosystems) and were analyzed using an EvoSep One liquid chromatography system connected to a timsTOF Flex mass spectrometer (Bruker) through a 15 cm x 150 µm, 1.5 µm C18 based column (EvoSep Biosystems, #EV1137). The LC was operated using the 30 samples per day method. Mass spectra was acquired using data independent acquisition parallel accumulation-serial fragmentation (diaPASEF) using a window schema defined and optimized in part by py_diAID [13]. Data processing was conducted through DIA-NN using standard settings [14] utilizing “FASTA digest for library-free search” approach, with deep learning-based spectra, RTs and IMs predication enabled. Neural network classifier was set to double-pass mode, with cross-run normalization disabled. All other analysis settings were maintained to factory settings and identification were set to 1% false discovery rate (FDR) for precursor and protein level.

### Statistical analysis

#### Amp-seq.

Demultiplexing of Amp-Seq data was performed with bcl2fastq software. The fastq files were analyzed CRISPResso2 software (https://github.com/pinellolab/crispresso2) with the following parameters: –min_paired_end_reads_overlap 8–max_paired_end_reads_overlap 300–ignore_substitutions -q 30 -w 1 -wc −3–plot_window_size 20–exclude_bp_from_left 15–exclude_bp_from_right 15. The counts of amplicons were fitted by a negative binomial regression model implemented in glm.nb function from the MASS R package. The logarithm of the total number of reads was used as the offset. The comparisons between groups were performed by using emmeans R package with the specified contrasts.

#### RNAseq.

Libraries were assessed using FastQC (v0.12.1), Qualimap (v2.2.2c) [15] and SAMtools stats (v1.18) [16]. Alignment was performed using STAR (version 2.7.2b) [17] with alignment against the human genome (GRCh38, Ensembl v105). Sequencing Quality control metrics were obtained using Qualimap (v2.2.2c) [15] and summarized using MultiQC (v1.17). Trimming of adapters was performed using NGmerge (v0.3) [18]. A human transcriptome index consisting of cDNA and ncRNA entries from Ensembl v105) was generated and gene abundances were obtained using Salmon (v1.1.0) [19]. The bioinformatics workflow was organized using Nextflow workflow management system (v20.10) [20] and Bioconda software management tool [21].

Differential gene expression analysis was performed using Dream (variancePatrition v1.32.2) [22]. Gene set enrichment analysis was performed with FGSEA (v1.28.0) (BioRxiv: https://doi.org/10.1101/060012) using Kyoto Encyclopedia of Genes and Genomes (KEGG; C2_CP:KEGG of the MSigDB collection v7.5.1 [23]). For plotting and data wrangling, Tidyverse (v2.0.0), PCAtools (v2.14.0) EnhancedVolcano (v1.18.0), and ComplexHeatmap (v2.18.0 [24]) were used. R 4.3.2 was used for downstream analyses.

#### Proteomics.

Un-normalized log2 transformed proteomic abundance values from DIA-NN were used to impute missing values where the proportion of missing values for a particular protein was less than or equal to 25%. In addition to this criterion, proteins were considered non imputable if they (a) contained 0% missingness and (b) contained constant values. Differential abundance analysis was performed by the Dream R package as in the RNAseq analysis. To avoid the bias of imputation, we performed the differential abundance analysis based on multiple imputed data sets and reported the average statistics.

## Results

### MCF7 and HepG2 share NHEJ dominance but differ in HR marker genes at protein level

The HRD score is a heuristic measure of HR deficiency and indicator of differences in editing efficiencies across cell types [9]. In this study, we investigated the molecular mechanisms linked to these differences focusing on two cell lines with different HRD scores: HepG2 and MCF7. HepG2 cells have an HRD score of 9 [12] and have been previously reported to undergo dramatic improvement in editing efficiency after inhibiting NHEJ [6]. Therefore, we considered HepG2 as cells whose precise KI efficiency is affected primarily by NHEJ dominance. On the other hand, MCF7 cells have an HRD score of 61 [12], indicating HR deficiency, and are selected to represent cells whose editing outcomes are affected by the dominant NHEJ and their HR deficiency (S1 Fig).

**Fig 1:**
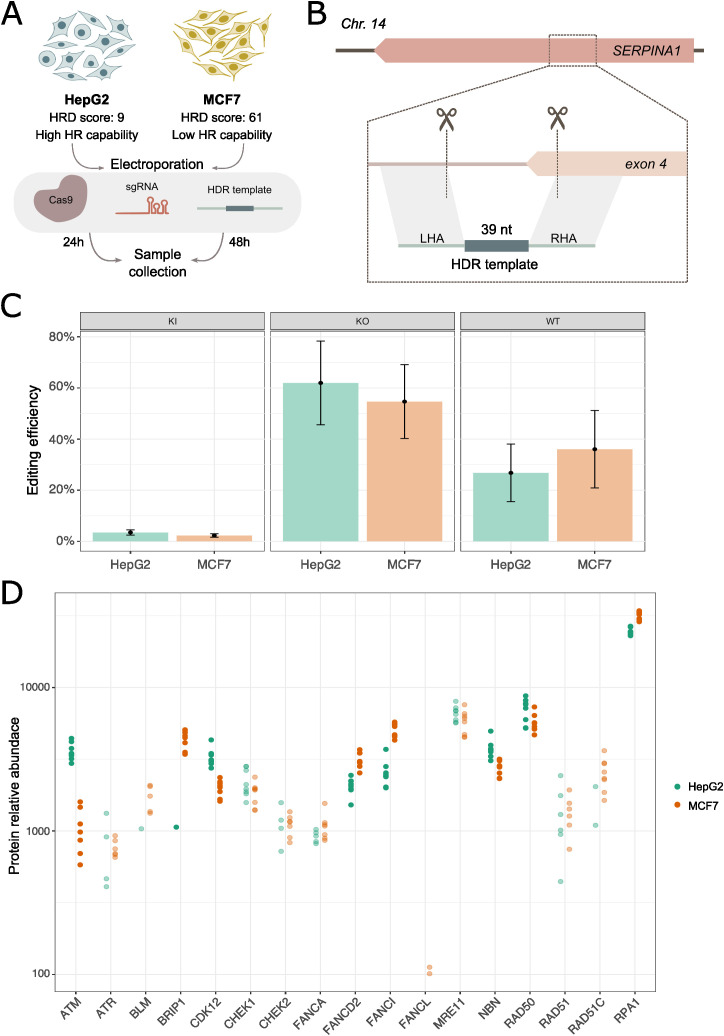
Experimental strategy overview and validation. **A)** Experimental overview for addressing CRISPR-Cas9 editing efficiency and OMICs generation for MCF7 and HepG2. **B)** Editing strategy to replace 88 bp by 39 bp at the *SERPINA1* locus, with a dual guide and a ssDNA template approach. **C)** Editing outcomes for *SERPINA1* locus in MCF7 and HepG2. **D)** Protein abundances of HR-related genes show differences in several HRD marker genes between MCF7 and HepG2.

To address KI efficiency in MCF7 and HepG2 cells, we targeted *SERPINA1* locus with spCas9, 2 guide RNAs and a single strand DNA (ssDNA) oligo serving as an HDR template in this study. *SERPINA1* encodes for α1-antitrypsin (A1AT), a circulating protease inhibitor secreted from the liver involved in protecting tissues from neutrophil elastase associated tissue damage [25]. We considered *SERPINA1* as a “safe-to-edit” locus as it is unlikely to interfere with cellular processes related to DNA repair and genomic stability. The desired editing outcome would replace 88 bp at *SERPINA1_exon 4* locus by an exogenous DNA sequence of 39 bp (Fig 1A, 1B). With this experimental setup, we addressed KI efficiency in both cell lines at the *SERPINA1* locus 48 hours after editing. KI efficiency was evaluated as the percentage of reads containing the precise KI using amplicon sequencing. Here, we observed similar editing outcomes in both cell types, with low KI efficiencies (HepG2 = 3.5%, MCF7 = 2.3%; P value = 0.7372) and high KO efficiencies (HepG2 = 62%, MCF7 = 55%; p-value = 0.3219), indicating NHEJ dominance in both cell types (Fig 1C).

Subsequently, we assessed whether the HRD scores indeed reflect phenotypic differences in HR related genes. For that, we performed proteomics analysis of HepG2 and MCF7 at baseline that revealed lower protein abundance in MCF7 cells for proteins representing multiple DDR pathways, such as ATM, BRIP1 and CDK12 (Fig 1D, S2 Table) than in HepG2. These observations support our assumptions that DNA repair in MCF7 is a result of the NHEJ and a deficiency in HR pathways, whereas in HepG2 is governed by a dominant NHEJ.

### CRISPR-editing induces different cell fates in MCF7 and HepG2

**Fig 2:**
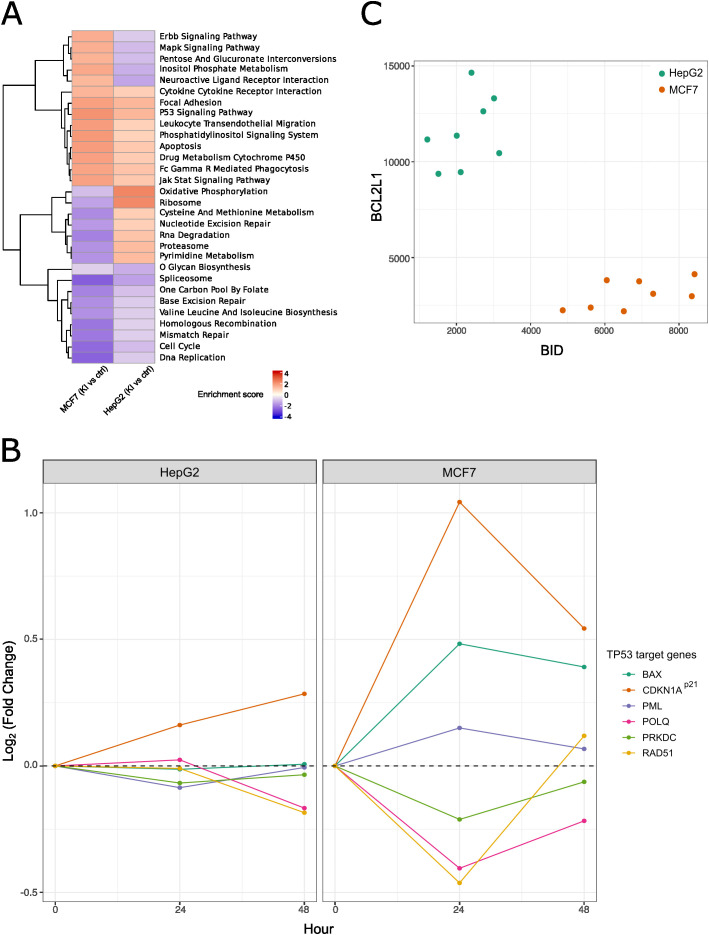
MCF7 and HepG2 activate different cell programs upon DNA damage. **A)** Pathway analysis of RNAseq data shows different pathway profiles between MCF7 and HepG2 upon electroporation of CRISPR-Cas9 reagents. **B)** MCF7 and HepG2 activate p53 signaling upon CRISPR-Cas9 editing but trigger different cellular outcomes, as shown by the differential expression of p53 target genes profile. **C)** Global proteomics shows differential protein abundance of proapoptotic BLM2 proteins (BID) between MCF7 and HepG2 at baseline, revealing MCF7 to be apoptotic primed.

Baseline proteomics analysis provided quantitative assessment of HR capabilities in HepG2 and MCF7. However, CRISPR-induced DDR involves a complex network of different and interconnected genes. To address whether these two cell lines respond to CRISPR-Cas9 induced DNA damage in a similar manner, we performed RNAseq analysis of MCF7 and HepG2 cells in which *SERPINA1* locus was targeted for editing following the same strategy as above. To capture transcriptional changes during and after precise DNA cut and KI resolution, our RNA-seq analysis focused on 24 and 48 hours after electroporation (Fig 1A, 1B). Our RNA-seq analysis confirmed that *SERPINA1* locus was perturbed in all edited cells, represented as a drop in reads covering *SERPINA1* locus (S2A Fig). In addition, we identified reads containing the accurate 39 bp knock-in in all edited samples (S2B Fig). Here, we identified 578 genes that responded differently between MCF7 and HepG2 cells 24 hours after electroporation of CRISPR components. In addition, we identified 72 genes that responded differently between these two cell types 48 hours after electroporation (S3 Table). Pathway analysis showed that MCF7 and HepG2 activate different transcriptional programs following delivery of CRISPR reagents. Moreover, we observed decreased expression of genes in DNA repair pathways (homologous recombination, base excision repair, and mismatch repair) and increased expression of genes involved in promoting apoptosis in MCF7 (Fig 2A). Following cell stresses such as DNA damage, p53 signaling plays an essential role in determining cell fates including cell-cycle arrest, apoptosis, or DNA repair [26], we further interrogated the expression levels of different p53 target genes leading to different cellular responses. We identified upregulation of CDKN1A (p21) and BAX in MCF7 (markers for cell cycle arrest and apoptosis, respectively), and downregulation of PRKDC, POLQ, and RAD51 (NHEJ, MMEJ and HR markers genes, respectively) in MCF7. In contrast, besides upregulation of CDKN1A (p21), we observed no expression changes in these marker genes in HepG2 cells, indicating a CRISPR-induced cell cycle arrest in HepG2 allowing for DNA damage repair (Fig 2B).

To answer why the same editing strategy leads to different cell fates, triggering apoptosis in MCF7 but not in HepG2, we interrogated the baseline protein abundance of pro-apoptotic and pro-survival marker genes within the B-cell lymphoma 2 (BCL-2) protein family, which are known for largely determine cell fate decisions between life and death [27]. Here, we observed higher levels of the pro-apoptotic BH3 interacting-domain death agonist (BID) protein in MCF7, but lower levels of the pro-survival protein BCL-XL (encoded by BCL2L1 gene) compared to HepG2 (Fig 2C). These observations suggest that MCF7 cells are primed for apoptosis, whereas HepG2 cells are primed for survival.

### Identification of potential target to improve editing outcomes in MCF7 cells

**Fig 3:**
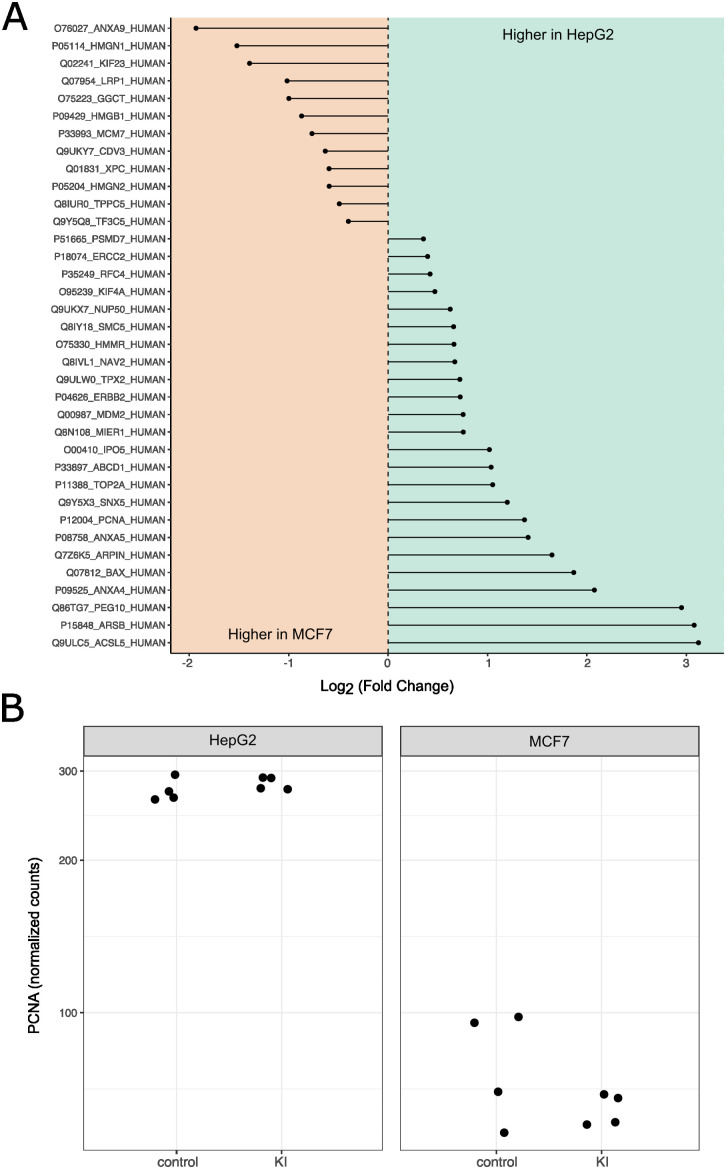
Identification of novel factors with potential to increase KI efficiency in MCF7. **A)** Identification of potential targets to modulate MCF7 editing outcome. **A)** Differentially expressed genes between MCF7 and HepG2 show protein level differences at baseline, including DDR and HR markers. **B)** Identification of PCNA as a candidate target to modulate MCF7 editing outcomes. Baseline levels of PCNA are higher in HepG2. Moreover, PCNA protein levels tend to decrease upon CRISPR-Cas9 editing.

Our omics analysis revealed that besides NHEJ dominance, precise KI of MCF7 cells is challenged by compromised HR repair and apoptotic priming. Next, we integrated the baseline proteomics and CRISPR-induced DDR transcriptomics to identify proteins with different abundance at baseline and different transcriptional responses to CRISPR editing between MCF7 and HepG2. Here, we identified 36 such proteins where the difference in abundance at baseline is directly linked to the differences in CRISPR-induced DDR outcomes between MCF7 and HepG2 (Fig 3A). Altogether, these observations suggest that modulating these DDR associated proteins may imply changes to the editing efficiencies.

To test this concept, we attempted to overexpress one of these 36 DDR proteins to increase the proportion of accurate CRISPR editing outcomes in MCF7. An overexpression candidate was chosen based on the following criteria: 1) must be underrepresented in MCF7 compared to HepG2; 2) should counteract apoptosis; 3) needs to promote entry into S phase to enable cells to repair DSBs with HR. Given these three requirements, we selected the scaffold proliferating cell nuclear antigen (PCNA) protein. At baseline, MCF7 has less PCNA protein than HepG2 (S3 Fig), and CRISPR-induced DDR leads to significant downregulation of PCNA transcription in MCF7 but not in HepG2 (Fig 3B). Moreover, PCNA is known for its indispensable role in maintenance of genomic integrity and DNA replication [28]. In addition, monomeric PCNA in the cytoplasm has been described as a key element to drive survival of non-proliferating cells [29].

### Transient overexpression of PCNA promotes precise knock-in efficiency in MCF7

**Fig 4:**
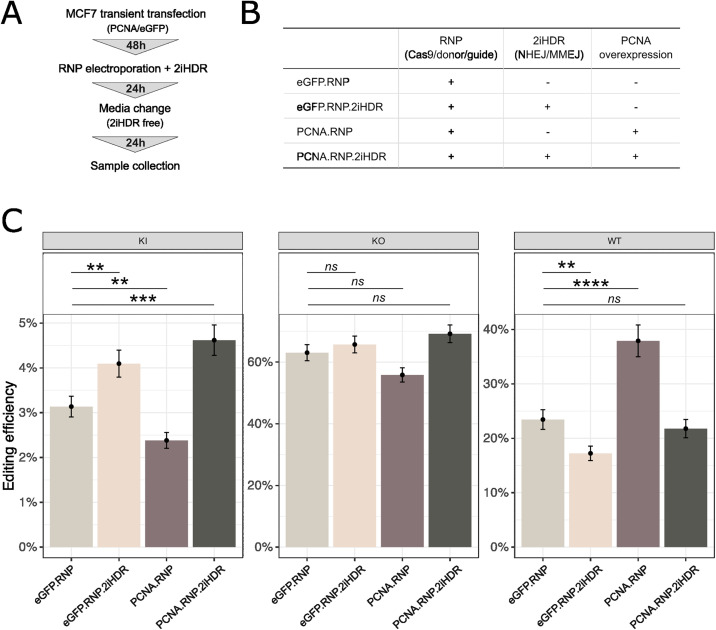
PCNA overexpression improves KI efficiency when used in combination with 2iHDR. **A)** Experimental overview for addressing editing outcomes in MCF7. **B)** Schematic representation of all combinations tested in MCF7. **C)** Editing outcomes for *SERPINA1* locus in MCF7 in all tested combinations. ** P < 0.01; *** P < 0.001; **** P < 0.0001; ns P > 0.05.

Based on our omics analysis, we elucidate why MCF7 cells are more challenging for precise KI compared to HepG2 cells and identified PCNA as a potential candidate to overexpress for improving editing efficiency in MCF7. To experimentally validate the role of PCNA in improving KI efficiency, we transiently transfected MCF7 with plasmids to overexpress PCNA, or eGFP as control, 48 hours prior electroporation of CRISPR editing reagents using the same editing strategy as described above (Fig 1A, 1B). In addition, to maximize the potential impact of PCNA on HR, we blocked NHEJ and MMEJ pathways by treating cells with DNA-PK and POL-Q inhibitors, here referred to as 2iHDR cocktail (AZD7648, PolQi1, and PolQi2 – at 1.5 µM each) [6] for 24 hours post electroporation (Fig 4A, 4B), and analyzed the editing outcome with amplicon sequencing. We observed that overexpressing PCNA in combination with 2iHDR resulted in 38.7% (P value = 0.0002) increase in KI efficiency for MCF7 cells compared to editing control (Fig 4C – PCNA.RNP.2iHDR vs eGFP.RNP). As a reference, addition of 2iHDR only resulted in 26.7% (P value = 0.0104) KI efficiency increase in MCF7 compared to editing control. PCNA overexpression enhances the effect of 2iHDR by 39.6% (P value = 0.0073) (Fig 4C). Interestingly, overexpression of PCNA in MCF7 cells without 2iHDR reduced overall editing efficiency, as shown by the 48% increase (P value < 0.0001) in wild-type reads (Fig 4C – PCNA.RNP).

## Discussion

The CRISPR-Cas system has revolutionized the field of molecular biology, providing the scientific community with a versatile tool to precisely and effectively modify DNA sequences in a wide range of *in vitro* and *in vivo* models. However, desired editing efficiency can vary dramatically depending on the underlying biology of cell models. To develop an experimental strategy that can improve efficiency of editing outcomes, it is critical to understand the underlying mechanisms and identify bottleneck protein targets that can be exploited. However, many genes are involved in DDR, making this task not trivial. Omics profiling provides a possibility to tackle this complexity as it measures thousands of molecules simultaneously. As a proof-of-concept example, we applied an omics approach to study two cell lines (HepG2 and MCF7) that both showed low KI efficiency. The low KI efficiency of HepG2 has been previously reported, and simultaneous inhibition of NHEJ and MMEJ pathways with 2iHDR treatment resulted in 6 times improvement in KI efficiency [6]. Since adding 2iHDR to HepG2 removes the NHEJ dominance, the reported dramatic improvement in KI efficiency indicates that HepG2 has fully functional HR repair. This is in line with its low HRD score (HRD score = 9). Therefore, HepG2 represents cells whose editing efficiency are mainly affected by NHEJ dominance. On the other hand, MCF7 is predicted to be less competent in HR repair as it has a large HRD score (HRD score = 61). Indeed, our baseline proteomics analysis showed that MCF7 had lower abundance of crucial HR direct repair proteins such as ATM and CDK12 compared to HepG2. These genes are known to play important roles in HR repair, such as the protein kinase ATM that activates several key proteins initiating HR repair [30], BRIP1 that interacts with BRCA aiding in DSB repair [31], and CDK12 that regulates the expression of DNA damage response genes essential for HR [32]. These observations suggest that precise KI edits are more challenging in MCF7 than in HepG2, as MCF7 is affected by both NHEJ dominance and HR deficiency.

Baseline proteomics analysis provided quantitative assessment of HR capabilities in HepG2 and MCF7. However, DSBs induced by CRISPR-Cas9 trigger DDR which involves a large number of interconnected genes. Unlike global proteomics which covers a portion of the genome, transcriptomic profiling enabled us to more comprehensively characterize the cell-specific DNA damage response at genome scale. Our transcriptomics analysis revealed that CRISPR-Cas9 editing induced activation of p53 signaling in both cell types but with emphasis on pathways for different cell fates in MCF7 (apoptosis) and HepG2 (cell cycle arrest). CRISPR-Cas9 is known to induce p53-mediated DDR [33], which transcriptionally regulates hundreds of genes that are involved in DNA damage repair, cell cycle arrest, apoptosis and senescence [34]. To answer why the same CRISPR-Cas9 editing induced apoptosis in MCF7 but not in HepG2, we assessed the abundance of BCL-2 family proteins that are known to largely determine cell fate decisions between life and death [27]. Here, we found that MCF7 cells had higher pro-apoptotic protein (BCL-2) and lower pro-survival protein (BCL-XL) at baseline compared to HepG2. This indicates that MFC7 is primed for apoptosis, whereas HepG2 is primed for survival. In fact, apoptotic priming plays a key role in determining cell fate upon p53 activation [35], and a reduction on apoptotic priming has been shown to be beneficial for precise KI efficiency [36]. These observations indicate that in addition to HRD score, apoptotic priming status is an additional factor to take into consideration when assessing editing ability.

Through our omics profiling, we now understand why precise KI in MCF7 is challenging: 1) as most mammalian cells, MCF7 have a powerful and dominant NHEJ; 2) they are HR compromised; and 3) are primed for apoptosis at baseline. By integrating proteomics and transcriptomics, we identified 36 proteins that were different between MCF7 and HepG2 at baseline that can differentiate the DNA damage response following CRISPR-Cas9 treatment. Among these proteins, we identified PCNA as a candidate for improving KI efficiency when ectopically expressed. PCNA acts as a sliding clamp to facilitate DNA replication and is indispensable for the maintenance of genomic integrity in actively growing cells [28]. We assumed that PCNA can reduce the propensity of apoptosis upon CRISPR-Cas9 editing since monomeric PCNA in the cytoplasm was found to drive survival of non-proliferating cells [29]. Moreover, PCNA was shown to promote cell cycle transition into S-phase, where the machinery for HR is active [37,38]. Since PCNA had a lower abundance in MCF7 compared to HepG2 at baseline, we hypothesized that overexpression of PCNA would reduce apoptosis and promote HR in MCF7. Interestingly, PCNA overexpression following CRISPR-Cas9 treatment did not increase KI efficiency. This may potentially be because PCNA overexpression primes MCF7 to utilize dominant DNA damage repair pathways for rapid and error-free resolution of Cas9-induced DSBs [39]. While reports have shown an increase in large deletions under NHEJ inhibition, blocking of PolQ and DNA-PK (2iHDR) has been shown to mitigate Cas9-related unwanted on-target effects, such as large deletions, presenting a potential advantage of 2iHDR over DNA-PK inhibition alone [6,40,41]. When we promoted homologous recombination pathways with PCNA overexpression and 2iHDR, we experimentally showed that the KI efficiency increased by 40% in MCF7 compared to editing control. Future studies will be required to characterize the genome-wide consequences of NHEJ/MMEJ inhibition and PCNA modulation on the repair of spontaneously occurring DSBs.

This proof-of-concept study aims to show how omics can aid develop editing strategy for hard-to-engineer cell models. While this study identified and evaluated PCNA overexpression in MCF7 cells, omics profiles from any source can inform choice of other candidate genes or even gene combinations for more efficient on-target editing outcomes, depending on the profile of the desired cell model. Indeed, this approach could be further extended to identify mutation status of key genes that may otherwise be adequately expressed (for example in DNA damage repair pathways) and replace them or overexpress with functional versions to improve editing outcomes. Recurrent efforts will be needed to get a more comprehensive understanding of mechanisms underlying precise knock-ins in additional cell backgrounds and how our findings for MCF7 cells could be translated into other cell models. A limitation of this study is that our characterization focused on gene and protein abundance only and cannot characterize post transcription modifications or kinetics, which plays an essential role in DDR. In summary, this study highlights the potential of employing multi-omics approaches to characterize our cell models and facilitate successful precise knock-in edits by manipulation of their basal state.

## Supporting information

S1 Fig Distribution of HRD score across different cell lines (CCLE database). Cell lines with HRD larger than 42 (dark grey) are considered HR-deficient. Dashed lines highlight HRD scores for HepG2 and MCF7, falling into 2 different HR categories.
(TIFF)

S2 Fig A) *SERPINA1* gene expression in control and edited cells (KI), showing a decrease in reads on edited cells due to CRISPR/Cas9 targeting. B) RNAseq reads aligning to HDR donor sequence are only present in the edited samples (KI).
(TIFF)

S3 Fig PCNA baseline protein levels relative to GAPDH protein. PCNA/GAPDH ratio reveals differences in PCNA baseline protein levels between MCF7 and HepG2 cell lines, with higher expression in HepG2.
(TIFF)

S1 Table Cell engineering reagents for precise knock-in at the *SERPINA1* locus, and primers used for validation of the insert. (XLSX)

S2 Table Differential abundance of HRD related proteins between HepG2 and MCF7 at baseline. (XLSX)

S3 Table Differential expression of genes between HepG2 and MCF7 at 48h after electroporation of CRISPR reagents. (XLSX)
