# Supplementary figures and images for: Omics-aided design genome editing strategy for challenging human immortalized cell models
Source: PLoS One. 2026 Feb 12;21(2):e0341124. doi: 10.1371/journal.pone.0341124 (PMC12900285; doi:10.1371/journal.pone.0341124)

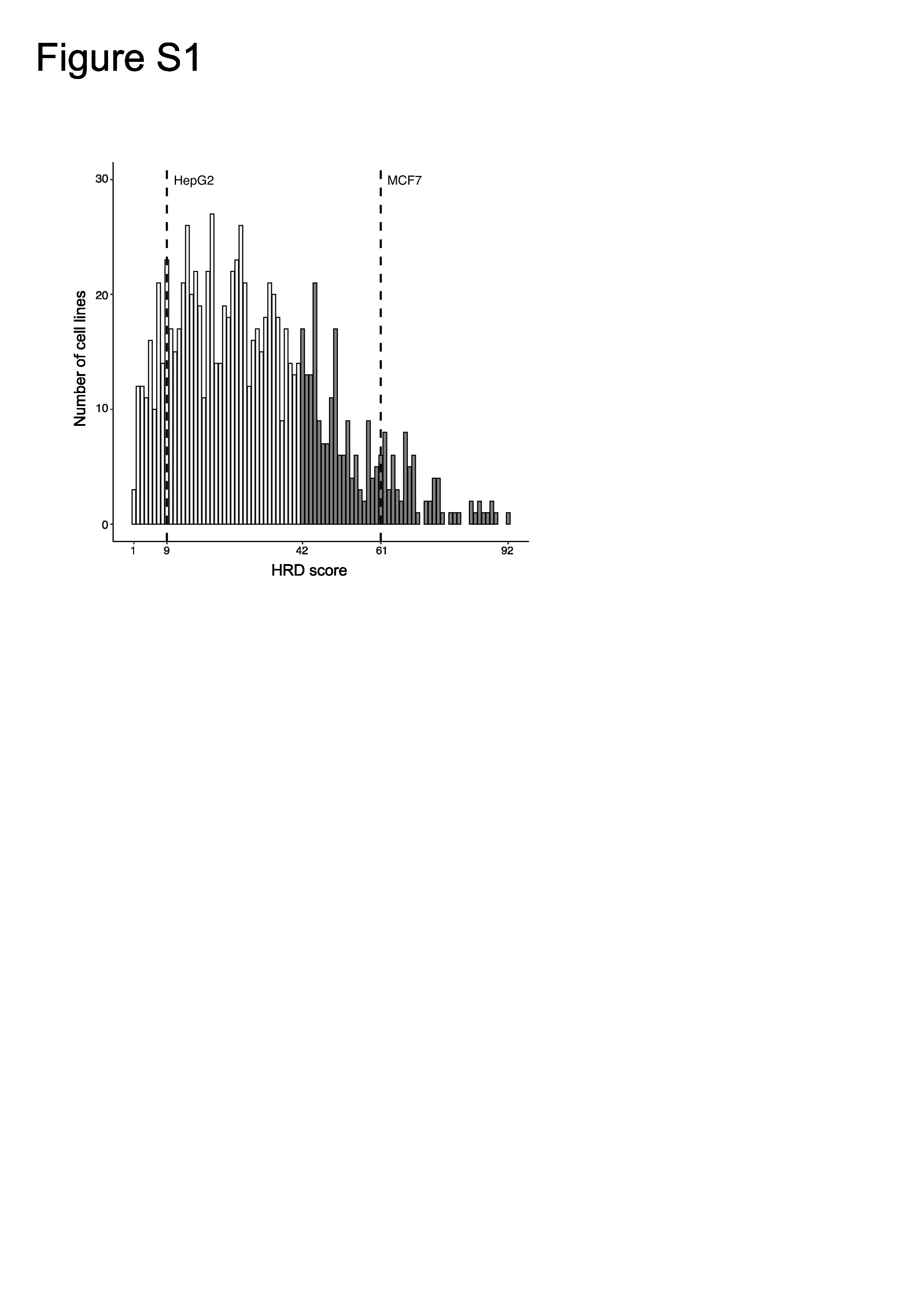

Supplement: S1 Fig — Cell lines with HRD larger than 42 (dark grey) are considered HR-deficient. Dashed lines highlight HRD scores for HepG2 and MCF7, falling into 2 different HR categories. (TIFF) [file pone.0341124.s001.tiff]

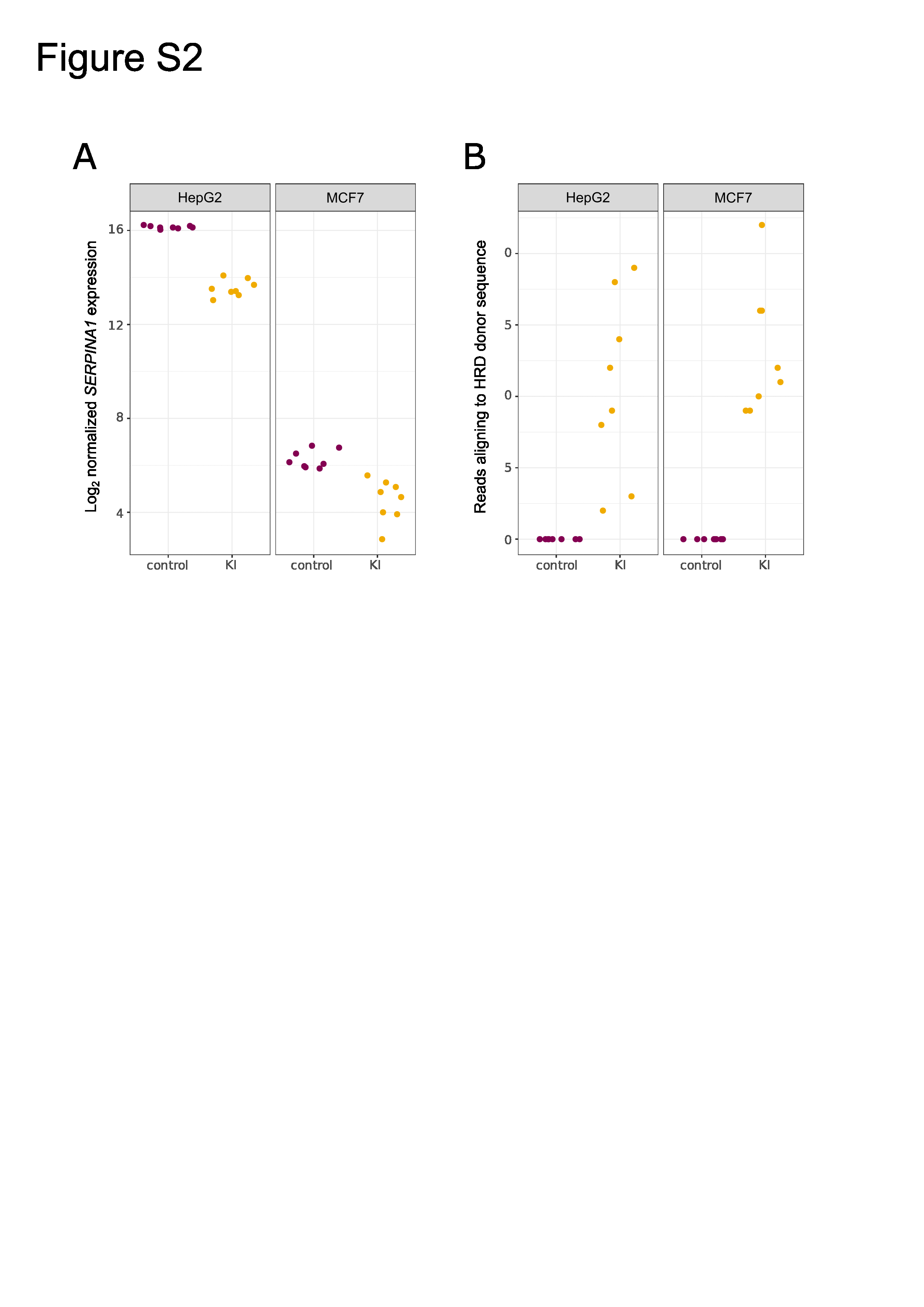

Supplement: S2 Fig — B) RNAseq reads aligning to HDR donor sequence are only present in the edited samples (KI). (TIFF) [file pone.0341124.s002.tiff]

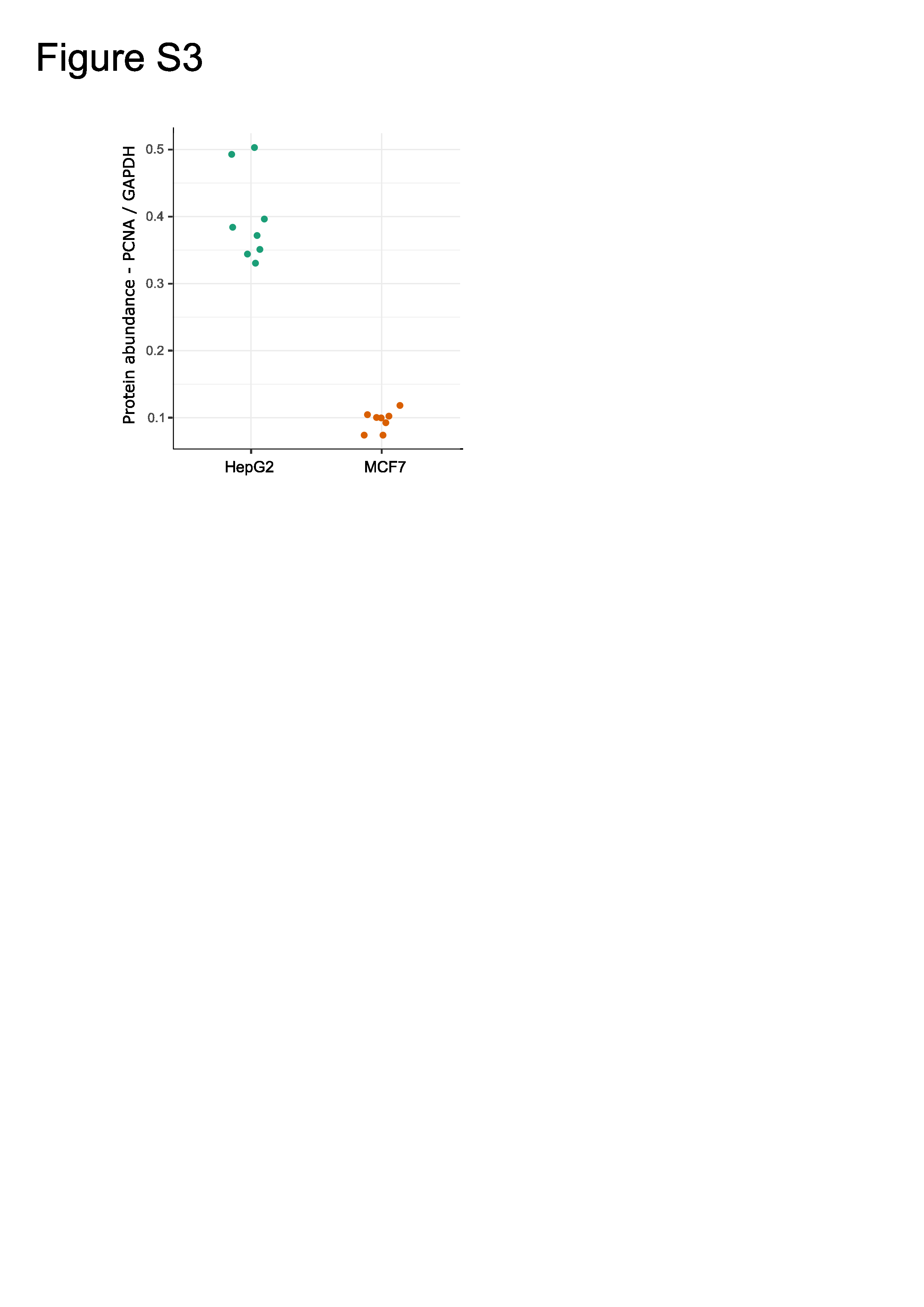

Supplement: S3 Fig — PCNA/GAPDH ratio reveals differences in PCNA baseline protein levels between MCF7 and HepG2 cell lines, with higher expression in HepG2. (TIFF) [file pone.0341124.s003.tiff]
